
JOURNAL INFORMATION
==============================
NLM Title Abbreviation: ScientificWorldJournal
Journal ID: ScientificWorldJournal
NLM Journal ID: 101131163
Journal ID: TSWJ

The Scientific World Journal

ISSN: 2356-6140
EISSN: 1537-744X
Publisher: Wiley

ARTICLE INFORMATION
==============================
PMCID: PMC6009753
PMID: 29973786
DOI: 10.1100/tsw.2002.12
Article ID: 629575
Article version: 1
Subjects: Short Report

Proteomic Analysis of Rough and Smooth Endoplasmic Reticulum

Paiement Jacques 1 *
Roy Line 2
Gilchrist Annalyn 2
Bell Alex 2
Kearney Rob 2
Thomas David Y. 3
Bergeron John J.M. 2
Département de pathologie et biologie cellulaire, Faculté de médecine, Université de Montréal, Québec, Canada
Department of Anatomy and Cell Biology, McGill University, Québec, Canada
Department of Biochemistry, McGill University, Québec, Canada
*Jacques Paiement: paiemej@patho.umontreal.ca
Publication date: 2002
Electronic publication date: 2002 Apr 9
Volume: 2
First page: 23
Last page: 24
Copyright: Copyright © 2002 Jacques Paiement et al.
Copyright year: 2002
License: This is an open access article distributed under the Creative Commons Attribution License, which permits unrestricted use, distribution, and reproduction in any medium, provided the original work is properly cited.
License URL: https://creativecommons.org/licenses/by/3.0/

Funding: Canadian Institutes of Health Research GSP36652

==============================
Advances in Gene Technology: The Genome and Beyond –
Structural Biology for Medicine (Proceedings of the 2002 Miami
*Nature Biotechnology* Winter Symposium)
*TheScientificWorld* 2002, 2(S2), 23–24
ISSN 1532-2246; DOI 10.1100/tsw.2002.12

### PROTEOMIC ANALYSIS OF ROUGH AND SMOOTH ENDOPLASMIC RETICULUM

Jacques Paiement[1], Line Roy[2], Annalyn Gilchrist[2], Alex Bell[2], Rob Kearney[2], David Y. Thomas[3], and John J.M. Bergeron[2]
[1]Département de pathologie et biologie cellulaire, Faculté de médecine, Université de Montréal, Québec, Canada; [2]Department of Anatomy and Cell Biology and [3]Department of Biochemistry, McGill University, Québec, Canada
paiemej@patho.umontreal.ca

**INTRODUCTION.** The membrane subcompartments of the endoplasmic reticulum (ER) are known to be continuous but yet complete structural and functional identity is maintained in each. This is achieved by membrane differentiation and maturation and likely involves sorting and transport that are mediated by different classes of vesicles and tubules. Cytosolic proteins and signalling molecules[1] probably assemble on the membranes to effect vesicle and tubule formation. Study of the ER using proteomic analysis should help define the molecular components involved in ER differentiation.

**METHOD.** ER membrane fractions enriched in rough and smooth microsomes were isolated by differential centrifugation and sucrose gradient sedimentation from rat liver homogenates[2]. For proteomic analysis, ER fractions were subjected to high salt wash treatment and were further biochemically fractionated into TX-114: soluble, detergent or insoluble phases. 1- and 2-D gel electrophoresis profiles were established for each of these fractions providing protein spots for transmembrane (TX-114 detergent), matrix (TX-114 insoluble), and cytosolic ER associated (TX-114 soluble/salt-wash supernatant) proteins. Candidate protein spots were cut out from gels and subjected to trypsin digestion. Membrane and protein treatments were previously described[3].

The extracted tryptic peptides were analysed automatically employing a CapLC-QtoF mass spectrometer in the tandem MS switching mode.

**RESULTS.** A complete proteomics analysis of the ER is underway with 2-D gel comparisons of rough and smooth ER well advanced. In addition, using 1-D gel separations, hundreds of protein identifications have been made by tandem mass spectrometry and unique novel proteins identified. Numerous proteins were identified as common to both rough and smooth ER but many proteins were found to be unique to either of these two ER subcompartments. Cytosolic proteins and signalling molecules were often found amongst the unique proteins and some of these have already been defined by published work to be involved in specific functions of the ER (i.e., 4). Greater than 75% of the proteins previously identified in a proteomic study of the outer membrane of the nuclear envelope[5] have been identified in our ER preparations.

**CONCLUSION.** The identification of known proteins associated with specific macromolecular structures such as ribosomes in particular ER fractions allowed us to assign novel proteins to distinct ER subcompartments. By being able to attribute the presence of these novel proteins to specific ER subcompartments we are now able to predict the possible involvement of multiprotein complexes in membrane differentiation and maturation processes in the ER.

**ACKNOWLEDGEMENT.** This work was supported by a grant (No. GSP36652) from the Canadian Institutes of Health Research.

**REFERENCES**

1. Kirchhausen, T. (2000) Natl. Rev. Mol. Cell Biol. 1, 187–198.
2. Roy, L., Bergeron, J.J.M., Lavoie, C., Hendriks, R., Gushue, J., Morré, D.J., Subramaniam, J.N., Hong, W., and Paiement, J. (2000) Mol. Biol. Cell 11, 2529–2542.
3. Bell, A., Ward, M.A., Blackstock, W.P., Freeman, H.N., Choudhary, J.S., Lewis, A.P., Chotai, D., Fazel, A., Gushue, J., Paiement, J., Palcy, S., Chevet, E., Lafrenière-Roula, M., Solari, R., Thomas, D.Y., Rowley, A., and Bergeron, J.J.M. (2001) J. Biol. Chem. 276, 5152–5165.
4. Lavoie, C., Chevet, E., Roy, L., Tonks, N.K., Fazel, A., Posner, B., Paiement, J., and Bergeron, J.J.M. (2000) Proc. Natl. Acad. Sci. U. S. A. 97, 13637–13642.
5. Dreger, M., Bengtsson, L., Schoneberg, T., Otto, H., and Hucho, F. (2001) Proc. Natl. Acad. Sci. U. S. A. 98, 11943–11948.
